# Anticancer properties of sulforaphane: current insights at the molecular level

**DOI:** 10.3389/fonc.2023.1168321

**Published:** 2023-06-16

**Authors:** Muhammad Asif Ali, Noohela Khan, Nabeeha Kaleem, Waqas Ahmad, Salem Hussain Alharethi, Bandar Alharbi, Hassan H. Alhassan, Maher M. Al-Enazi, Ahmad Faizal Abdull Razis, Babagana Modu, Daniela Calina, Javad Sharifi-Rad

**Affiliations:** ^1^ Department of Food Science and Human Nutrition, University of Veterinary & Animal Sciences, Lahore, Pakistan; ^2^ Department of Nutrition Sciences, Rashid Latif Medical College, Lahore, Pakistan; ^3^ Department of Biological Science, College of Arts and Science, Najran University, Najran, Saudi Arabia; ^4^ Department of Medical Laboratory Science, College of Applied Medical Sciences, University of Ha’il, Hail, Saudi Arabia; ^5^ Department of Clinical Laboratory Science, College of Applied medical Sciences, Jouf University, Sakaka, Saudi Arabia; ^6^ Department of Medical Laboratory Sciences, College of Applied Medical Sciences in Al-Kharj, Prince Sattam Bin Abdulaziz University, Al-Kharj, Saudi Arabia; ^7^ Department of Food Science, Faculty of Food Science and Technology, Universiti Putra Malaysia, Serdang, Selangor, Malaysia; ^8^ Natural Medicines and Products Research Laboratory, Institute of Bioscience, Universiti Putra Malaysia, Serdang, Selangor, Malaysia; ^9^ Department of Biochemistry, Faculty of Science, University of Maiduguri, Maiduguri, Borno, Nigeria; ^10^ Department of Clinical Pharmacy, University of Medicine and Pharmacy of Craiova, Craiova, Romania; ^11^ Facultad de Medicina, Universidad del Azuay, Cuenca, Ecuador

**Keywords:** sulforaphane, isothiocyanate, anticancer mechanisms, cytotoxicity, apoptosis

## Abstract

Sulforaphane (SFN) is an isothiocyanate with multiple biomedical applications. Sulforaphane can be extracted from the plants of the genus *Brassica*. However, broccoli sprouts are the chief source of sulforaphane and are 20 to 50 times richer than mature broccoli as they contain 1,153 mg/100 g. SFN is a secondary metabolite that is produced as a result of the hydrolysis of glucoraphanin (a glucosinolate) by the enzyme myrosinase. This review paper aims to summarize and understand the mechanisms behind the anticancer potential of sulforaphane. The data was collected by searching PubMed/MedLine, Scopus, Web of Science, and Google Scholar. This paper concludes that sulforaphane provides cancer protection through the alteration of various epigenetic and non-epigenetic pathways. It is a potent anticancer phytochemical that is safe to consume with minimal side effects. However, there is still a need for further research regarding SFN and the development of a standard dose.

## 1 Introduction

Cancer is one of the most prevalent and fatal diseases throughout the globe. As per the World Health Organization, in 2020, cancer is the sole reason for the death of more than 10 million individuals (1). For the treatment of cancer, various medical treatments are applied, including chemotherapies and radiotherapies. Besides their potential benefits, such treatments also impose detrimental effects on various organs of the body (2). The consumption of phytochemicals must be incorporated into our diet to benefit from their anticancer properties. Since the beginning, cruciferous vegetables are known for their anticancer properties. Unfortunately, these are never ingested in sufficient amounts that can alter the health biomarkers. Cruciferous vegetables are rich in several nutrients and bioactive components, especially isothiocyanates that provide various health benefits (3). The isothiocyanates are the hydrolytic metabolites of glucosinolates, and examples include allyl isothiocyanate (AITC), benzyl isothiocyanate (BITC), phenethyl isothiocyanate (PEITC), and sulforaphane (SFN) (4, 5). The most important biological active isothiocyanate is sulforaphane, or simply SFN, which is potent in providing numerous health benefits by modulating epigenetic and non-epigenetic mechanisms. It did not receive much attention until 1992, even though it was first identified and isolated in 1959 (6). This updated review aimed to summarize and highlight the mechanisms behind the anticancer potential of sulforaphane.

## 2 Review methodology

This comprehensive review aims to provide updated information on the anticancer mechanisms of sulforaphane. The most relevant and recent data regarding the anticancer activity of sulforaphane were obtained through an online search of the following scientific databases—PubMed/MedLine, Scopus, Web of Science, and Google Scholar—using the next MeSH terms: “Brassica/chemistry”, “Chemoprevention”, “Isothiocyanates/metabolism”, “Isothiocyanates/pharmacology”, “Isothiocyanates/therapeutic use”, “Neoplasms/drug therapy”, “Neoplasms/prevention & control”, “Biological Products/therapeutic use”. The chemical structure has been validated using Pubchem and the taxonomy of the plant according to WorldFloraOnline (7, 8). The most representative data regarding the anticancer activities of sulforaphane are summarized in a schematic diagram and tables.

## 3 Bioavailability of sulforaphane and applications of nanotechnologies in improving its bioactivity

The dietary sources of SFN include mainly the plants (especially cruciferous vegetables) of the genus *Brassica* such as broccoli, brussels sprouts, kale, and cauliflower (3). As per a recent review, broccoli sprouts are the chief source of sulforaphane and are 20 to 50 times richer than mature broccoli as they contain 1,153 mg/100 g, whereas the concentration of SFN in mature broccoli is 44–171 mg/100 g. Sulforaphane is significantly effective as it is readily available in blood because of its high bioavailability (80%). The higher bioavailability is due to its lighter molecular weight in comparison to other polyphenols (6, 9). Recently, the trend of nanoencapsulation of various bioactive components to gain maximum benefits is increased. Since the last century, a lot of progress has been made in the field of nanotechnology. Advancement in research has made it possible for the development of polymeric biodegradable nanoparticles for better results and controlled/targeted delivery of drugs. Longhai Piao et al. likewise worked on the nanoencapsulation of sulforaphane using a triblock copolymer PCL-PEG-PCL, *i*.*e*., poly(caprolactone)-poly(ethylene glycol)-poly(caprolactone). The results revealed that the PCL-PEG-PCL-encapsulated SFN enhanced the cancer cell apoptosis more efficiently than the free sulforaphane in the *in vitro* trial. Additionally, the study validated that the SFN encapsulation with triblock copolymer was safe and can be used for effective and targeted sulforaphane delivery to cancerous cells (10). A study on the effect of basil seed gum-encapsulated broccoli sprout extract disclosed that encapsulation helped in the controlled release of sulforaphane, which is more effective in the treatment of cancer than free sulforaphane. Furthermore, the data also revealed that the release of SFN from the micelles was more in intestinal-stimulated conditions compared to the gastric-stimulated condition (11). Similarly, the results of an investigation conducted by Lucía Yepes-Molina et al. showed that the encapsulated sulforaphane had more stability. Additionally, in the *in vitro* trial, the investigators discovered that the SFN-rich membrane vesicles were potent in reducing the inflammatory markers on HL-60 cell linings. Another group of investigators worked on the encapsulation of sulforaphane in broccoli membrane vesicles (BM vesicles). The study evaluated the capability and potential of encapsulated sulforaphane on SK-MEL-28 (melanoma) cell linings. The *in vitro* analysis revealed that BM vesicles containing SFN had high absorption and metabolism in cancerous cells. Furthermore, encapsulated sulforaphane efficiently reduced cancer and its markers (12).

## 4 Phytochemistry of sulforaphane and its derivatives

Sulforaphane is chemically a product of a 4-(methylsulfonyl) butyl group that is attached to a nitrogen atom (6, 13, 14). SFN is a secondary metabolite produced as a result of the hydrolysis of glucoraphanin (a glucosinolate) by the enzyme myrosinase (6, 9, 15). Because sulforaphane has gained a lot of attention due to its potential against numerous diseases, especially cancer, scientists are interested in developing its derivatives which could be an important discovery in the world of medicine for cancer treatment. The group of Prachi Heda et al. had made some progress in this regard by synthesizing sulforaphane derivatives including SFN-glutathione, SFN-cysteine-glycine, SFN-cysteine, SFN-N-acetylcysteine, PEITC, and sulforaphane. Out of all these SFN derivatives, phenethyl isothiocyanate was found to be the most effective due to its high bioavailability, gastric absorption, and blood–brain barrier permeability. PEITC was involved in the inhibition of human S-adenosylmethionine decarboxylase (a lyase), p38alpha (a transferase), heme-oxygenase 1 (an oxidoreductase), and human cytochrome P450 (an oxidoreductase) activation. Furthermore, the downregulation of these enzymes exhibited a reduction in cancerous cell proliferation and angiogenesis and other cancer-progressing mechanisms (16). A review paper also unveiled the similar anticancer properties of sulforaphane and its derivatives, including sulforaphane and phenethyl isothiocyanate (17). Another *in vitro* study also disclosed that benzyl sulforaphane (a derivative of SFN) is more efficient in preventing and treating liver carcinoma by restraining the Akt/MAPK pathways and triggering the Nrf2/ARE signaling pathways (18). Similarly, Kun Hu et al. derived a series of SFN derivatives and evaluated the potential of these derivatives in an *in vitro* study on five different cell linings isolated from liver hepatocellular carcinoma (HepG2), human breast adenocarcinoma (MCF-7), human lung adenocarcinoma (A549), human neuroblastoma (SH-SY5Y), and human colon cancer (HCT-116). The data revealed that the sulforaphane derivative synthesized by introducing the benzyl group at the side chain of sulforaphane (benzyl sulforaphane) showed enhanced anticancer activity than any other derivative and SFN itself. The benzyl derivative of sulforaphane triggered cell cycle arrest apoptosis and Nrf2 activation more efficiently than SFN (19).

## 5 Anticancer mechanisms of sulforaphane

### 5.1 Cellular and molecular implications

**Figure 1:**
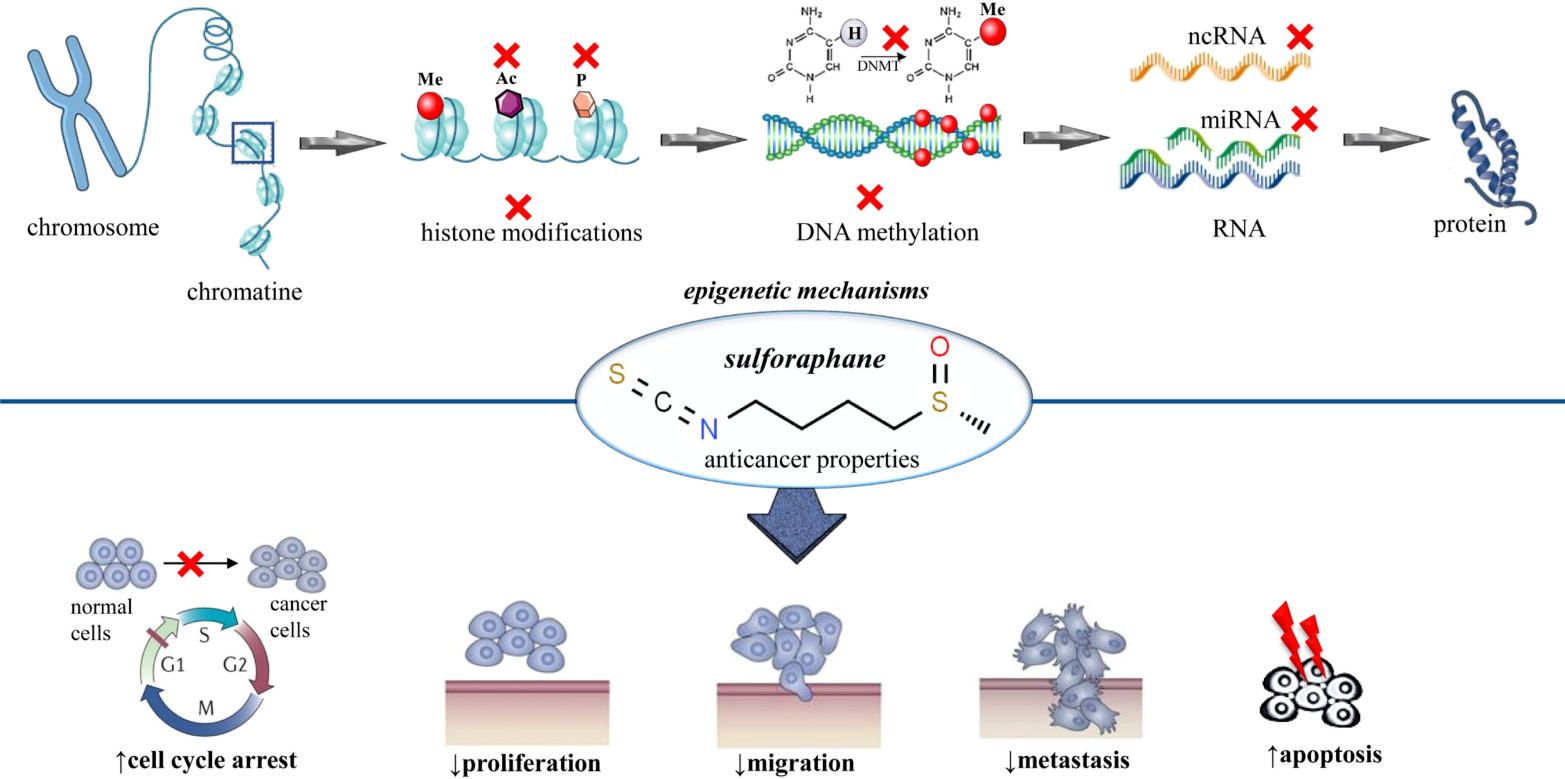
Summarized scheme regarding epigenetic modifications of sulforaphane in cancer. At the cellular level, sulforaphane shows anticancer activity by altering the epigenetic mechanisms. These epigenetic mechanisms involve histone acetylation, histone phosphorylation, DNA methylation, and modulation of noncoding RNAs. x, inhibition; Ac, acetylation; P, phosphorylation; Me, methylation; DNA, deoxyribonucleic acid; RNA, ribonucleic acid; DNMT, DNA methyltransferase.

Since the discovery of sulforaphane, many scientific studies outlining its health benefits have been published; it is a biologically active phytochemical that has many biological properties, such as anti-inflammatory, anticancer, cardioprotective, antioxidative, cytoprotective, DNA protective, and antimicrobial properties. Additionally, it is also a potent immune booster and detoxifier (9, 14). **Table 1** presents all the beneficial effects of sulforaphane proven in various research articles. Although SFN shows various properties, the most important property is its potential against cancer. SFN protects against cancer by inhibiting cancer cell proliferation, arresting the cell cycle, and enhancing the process of apoptosis (24–26). Sulforaphane provides cancer protection via the alteration of several epigenetic and non-epigenetic mechanisms; this was demonstrated in the case of many types of cancer. Sulforaphane stalls the activity of the enzyme histone deacetylase (HDAC) in cancerous cells. The inhibition of histone deacetylase is important in cancer prevention as it enhances several mechanisms such as apoptosis and cell cycle arrest (50–54). Furthermore, SFN also halts histone phosphorylation by enhancing the phosphatases, especially PP1β and PP2α (25, 55, 56). The epigenetic modulation of sulforaphane on breast cancer was assessed in another study. The results disclosed that there was a significant reduction in histone deacetylase activity and the cell proliferation marker (Ki-67) (57). Another study revealed that SFN downgraded processes like cell proliferation, the activity of histone deacetylase, and cell growth. Furthermore, it reduced the expression of various receptors such as estrogen receptor-α and human epidermal growth factor receptor-2 in cancerous cell lining (58) (**Figure 1**).

**Table 1:** Summary of sulforaphane biological activities.

Bioactivity	Mechanism	Reference
**Anti-inflammatory**	↓proinflammatory markers, ↓NF- kB	(20–23)
**Anticancer**	↑cell cycle arrest, ↓metastases, ↓angiogenesis ↑apoptosis, ↑Nrf2	(24–26)
	Protects the DNA, ↑ histone deacetylase	(27–30)
	↑Nrf2 antioxidant signaling cascade	(29, 31–34)
**Antioxidant**	↑antioxidant defense, ↑GSH	(22, 35, 36)
**Cardioprotective**	↓oxidative stress ↑TRxRS, ↑GR, ↑GSTs, ↑NQO1	(36–38)
**Cytoprotective**	The antioxidative potential of sulforaphane enhanced the production and activity of cytoprotective proteins to help protect the cell lining ↑cellular defense mechanisms, ↑detoxification, ↑redox reactions, ↑Nrf2	(32, 33)
**Immunostimulant**	Immune booster, ↑NK cell activity	(22)
**Antimicrobial**	↓growth of various gram-positive and gram-negative bacteria by preventing pyocyanin production, biofilm formation, and quorum sensing highly effective against *Helicobacter pylori*	(39–42)
**Anti-carcinogenesis**	Nrf2 and TrxRs have a dual role in cancer by protecting against oxidative stress. However, overactivation can promote tumor growth and may cause chemoresistance. Therefore, sulforaphane dosage must be selected carefully	(43–49)

**↑,** increase; ↓, decrease; Nrf2, nuclear transcription factor; TRxRS, thioredoxin reductase; GSH, glutathione; GR, glutathione reductase; GSTs, glutathione-S transferase; NADPH, nicotinamide adenine dinucleotide phosphate; NQO1, NAD(P)H quinone oxidoreductase; NK cells, natural killer cells.

DNA methyl transferases (DNMTs) are a family of enzymes that catalyze the transfer of the methyl group to the DNA. The suppression of these enzymes enhances the process of apoptosis and cell cycle arrest. SNF actively downregulates the function of DNMTs (especially DNMT1 and DNMT3B) and protects against cancer (25, 54, 59, 60) (**Figure 1**). Noncoding RNA is a molecule that does not need to be translated into protein to perform its functions. There are several different types of ncRNA, but miRNA (microRNA) and lncRNA (long noncoding RNA) are of great importance. These ncRNAs are usually involved in the regulation of numerous cell functions such as apoptosis, proliferation, and differentiation. The upregulation or downregulation of ncRNA plays a vital role in controlling the progression of cancer. Sulforaphane is one of those bioactive components that can suppress the growth of cancerous cells by regulating these ncRNAs. Various studies validated that SFN exhibits anticancer properties by halting as well as by enhancing the expression of a wide range of miRNAs and lncRNAs (25, 60–63). A study conducted by Scott R. Baier et al. likewise unveiled the off-target effects of SFN. The results were in favor of the hypothesis, *i*.*e*., SFN not only targets the cancer suppressor genes but also stimulates the transcriptional activity of long terminal repeats. The study also confirmed that the consumption of broccoli sprouts causes the inhibition of histone acetylation (64) (**Figure 1**).

Nuclear factor erythroid 2-related factor 2 (Nrf2) is a transcription factor that regulates the cellular defense mechanism (65). The activation of Nrf2 channels the cascade of antioxidants and exerts the anticancer effect by causing a reduction in cell transformation and development. SFN decreases cancer proliferation by enhancing the expression of Nrf2. Additionally, sulforaphane suppresses the expression of NF-_K_B that ultimately downregulates the inflammatory markers (25, 29, 31–34, 66, 67). The group of Julie E. Bauman studied the chemopreventive potential of sulforaphane by using preclinical models. The results of an *in vitro* model revealed that sulforaphane enhances the activation of Nrf2 signaling in normal mucosal epithelial cells and head and neck squamous cell carcinoma cell lines. The SFN extract was found to be highly effective in blocking the activity of inactivation of an oncogenic factor pSTAT3. However, in the outcomes of the *in vivo* model, SFN notably decreased the size and incidence of 4-nitroquinoline-1-oxide (a carcinogen) which induced oral cancer in mice (68). Several studies confirmed that sulforaphane provides cancer protection by inhibiting the proliferation of cancerous cells and downgrading the process of angiogenesis. To investigate this potential, Prayag et al. conducted an *in vitro* study. The results disclosed that sulforaphane notably increased the antioxidant cascade and enhanced the activity of natural cell killers by increasing the expression of MHC class I chain-related proteins A and B (69).

### 5.2 The hormetic effect of sulforaphane

Hormesis is a phenomenon characterized by a biphasic dose or concentration–response, where low doses or concentrations of a compound can stimulate a beneficial effect, while high doses or concentrations can lead to inhibitory effects (70).

This means that, at low doses, a compound may have a stimulatory effect, while at high doses, it may have an inhibitory effect. The review of Calabrese et al. explained this hormetic effect of sulforaphane and revealed that SFN induces hormetic dose responses in studies through the upregulation of the Nrf2/ARE pathway. This biphasic response is well integrated, concentration dependent, and specific to targeted cell types. The hormetic response of sulforaphane can decrease the incidence and severity of various human-related pathologies, and it has also been found to be involved in the enhancement of stem cell proliferation. Similar hormesis-based chemoprotection has been reported with other dietary supplements such as curcumin, ginkgo biloba, ginseng, green tea, and resveratrol. The activation of the Nrf2 (nuclear factor erythroid-derived 2)/ARE (antioxidant response elements) pathway is likely a principal and underlying mechanism of hormesis, which can limit age-related damage, numerous disease processes progression, and induction of toxicities due to chemicals and radiations (70).

## 6 Synergic anticancer effect of sulforaphane in combination with other chemotherapeutic agents

Sulforaphane can also be used with conventional cancer treatments to achieve an enhanced effect. The group of Małgorzata Milczarek shed light on the development of new combinations by conducting an *in vitro* study. The data unveiled that the combination of sulforaphane and fluorouracil is more effective in enhancing the process of apoptosis than fluorouracil alone. In the *in vitro* analysis, it was confirmed that combination arrests the cancerous cell cycle in the S-phase in the colon cell linings, especially HT-29 and Caco-2 (71). Similarly, the combination of sulforaphane with gefitinib (a potent drug used for the treatment of lung cancer) also showed a marked increase in the inhibition of cell proliferation and expression of numerous factors in comparison to both agents alone (72). Recently, another study disclosed that a combinatory regimen of SFN with allyl isothiocyanate is more effective in cancer prevention compared to a single treatment. The synergism between these two compounds enhanced the arrest of the cell cycle and the programmed cell death. In addition to this, the investigators found that the combined therapy also caused an increase in the downregulation of cell migration (73). Similarly, another research supported the notion that both luteolin and SFN together provide enhanced anti-inflammatory properties than alone. The dose-dependent inhibition of NO by sulforaphane was 51% and by luteolin was 46%, respectively. However, the combined therapy demonstrated inhibited nitric oxide production by 56%, proving that the combination led to an increase in the reduction of inflammatory markers, especially the decrease in nitric oxide production (74).

## 7 Clinical studies

Broccoli is rich in glucoraphanin which is a precursor of sulforaphane. To study the effect of sulforaphane, a randomized control trial on the prevention of prostate cancer was designed by a group of investigators. The study showed that sulforaphane significantly reduced the progression of prostate cancer and disease severity after the broccoli soup intervention for at least 1 year by altering the gene expression (75). Melanoma is a form of skin cancer that originates from melanocytes (pigment-producing cells). Several studies inferred that the presence of atypical nevi is one of the key risk factors for melanoma. Therefore, in melanoma patients, Shawn Tahata et al. explored the effect of the oral administration of broccoli sprout extract at three different dose levels (50, 100, and 200 µmol). The study inferred that sulforaphane significantly reduced the levels of proinflammatory cytokines in plasma and increased the tumor suppressor decorin in tissues, thus preventing the risk of melanoma (76). Another randomized double-blinded study disclosed that sulforaphane is highly effective in reducing serum prostate-specific antigen (PSA) levels. Serum PSA levels are usually high in men suffering from prostate cancer after radical prostatectomy. The study inferred that the administration of 60 mg SFN in the form of a tablet significantly reduced the PSA progression after at least 3 months of treatment (77). A recent study suggested that sulforaphane is highly beneficial in preventing ulcerative disease by inhibiting the growth of *Helicobacter pylori.* As per the group of Akinori Yanaka, the consumption of broccoli sprouts consecutively for 2 months can downgrade the colonization of the bacterium. This poses a cancer-preventive effect because of the reduction in oxidative stress caused by *H. pylori* (78). Additionally, several ongoing and completed clinical trials aim to investigate the chemoprotective and preventive effects of sulforaphane. The investigations also revealed that sulforaphane can help prevent and reduce the risk of cancer via modulating the gene expression by downregulating and upregulating various pathways in cells. However, there is still a need for further investigation on the sulforaphane cancer protective/preventive potential and mechanisms (57, 79–84). **Table 2** shows a summary of the representative data regarding the clinical studies with sulphoraphane.

**Table 2:** Clinical studies with sulphoraphane.

Study subjects	Type of study		Country	Enrolled subjects	Type of intervention		Status/phase	Objectives/main findings	Refs
Men on active surveillance for prostate cancer	Randomized control trial		United Kingdom	199 patients	Glucoraphanin-rich broccoli soup for 12 months		Completed	SFN significantly reduced the progression of prostate cancer and disease severity after the broccoli soup intervention for at least 1 year by altering the gene expression	(75)
Melanoma patients with multiple atypical nevi	Randomized control trial		United States of America	17 patients	Broccoli sprout extract at three different dose levels (50, 100, and 200 µmol) for 28 days		Completed	SFN prevented the risk of melanoma by reducing plasma proinflammatory cytokines and by enhancing the tumor suppressor decorin in tissues	(76)
Men with biochemical recurrence after radical prostatectomy	Double-blinded, randomized, placebo-controlled multicenter trial		France	78 patients	60 mg of oral sulforaphane (two tablets comprising of 10-mg sulforaphane each for three times a day) for 6 months		Completed	SFN is highly effective in reducing the serum PSA levels	(77)
*Helicobacter pylori-positive* volunteers	Randomized control trial		Japan	50 participants	70 g/day of glucoraphanin-rich 3-day-old germinated broccoli sprouts (Broccoli Super Sprout) for 8 weeks		Completed	The consumption of broccoli sprouts consecutively for 2 months can downgrade the colonization of *Helicobacter pylori* and provide a cancer-preventive effect due to the reduction in oxidative stress caused by *H. pylori*	(78)
Former smokers	Randomized clinical trial		United States of America	67 participants	Four tablets of sulforaphane two times per day (each dose comprising of 120 μmol of sulforaphane)		Active, not recruiting	The study aims to investigate that SFN can improve, maintain, or worsen the condition of former smokers who are at a high risk of developing cancer due to their smoking habit. Furthermore, the study also seeks to examine the potential of sulforaphane in reversing the changes in lung cells that are associated with cancer development	(84)
Men with recurrent prostate cancer	Phase II clinical trial		United States of America	20 patients	Sulforaphane-rich extracts (200 μmol/daily) for 20 weeks		Completed	The investigation revealed that using the SFN extract did not result in significant PSA declines in most of the patients with recurrent prostate cancer. Despite this, the treatment was found to be safe and had some positive effects on PSADT modulation. However, there is still a need for further investigation	(79)
Men scheduled for a prostate biopsy aged ≥21 years	Double-blind, randomized controlled trial		United States of America	98 participants	Sulforaphane-rich broccoli sprout extract capsules twice a day for 4 weeks (each capsule consists of 200 µmol of sulforaphane)		Completed	The supplementation of broccoli sprout extract is correlated with the modulation of gene expression. The study also disclosed that there was no significant difference in HDAC activity and the biomarkers of prostate cancer	(83)
Female patients of age greater than 18 years diagnosed with DCIS on core or incisional/excisional biopsy scheduled for definitive surgery	Phase II double-blind, randomized clinical trial		United States of America	34 participants	100 µmols of sulforaphane from broccoli sprout dissolved in 150 ml mango juice once a day for 14 days		Completed	The investigators aim to study the effect of the intervention on breast cancer-related factors. Furthermore, the investigators want to determine the potential of SFN in the enhancement of protective enzyme levels in breast tissue and the acceptability of the treatment among participants	(81)
Male patients of age 18–80 years scheduled for template biopsy of the prostate as part of routine investigation or staging for prostate cancer	Double-blind, randomized clinical trial	United Kingdom		40 participants	1. Two Allin capsules once a day for 4 weeks 2. Sulforaphane capsules once a day for 4 weeks	Completed		The study aims to investigate the relationship between the ingestion of allin, sulforaphane, and prostate metabolism.	(82)
Male patients between 40 and 75 years old with low- or intermediate-grade prostate cancer have a PSA level <20 ng/ml and have chosen radical prostatectomy, brachytherapy, or active surveillance as their main treatment	Randomized clinical trial	United States		45 participants	Sulforaphane-rich broccoli sprout extract (100 mol of sulforaphane, every other day for 5 weeks)	Completed		The investigation revealed the changes in phase II enzyme expression, levels of blood F2 isoprostane, levels of prostate tissue 8-hydroxy-2’-deoxyguanosine (8OHdG), levels of serum DHT (dihydrotestosterone) and testosterone, and levels of serum 3-alpha-diol gluconate (3α-DG)	(80)
Women with abnormal mammogram findings were scheduled for breast biopsies	Double-blind, randomized controlled trial	United States of America		54 participants	250 mg of a broccoli seed extract containing glucoraphanin as precursor to sulforaphane	Completed		The study disclosed that glucoraphanin supplementation for 2–8 weeks does not cause any adverse effects but may not be sufficient for producing changes in breast tissue tumor biomarkers. Future studies employing larger sample sizes should evaluate alternative dosing and duration regimens to inform dietary SFN strategies in breast cancer chemoprevention	(57)

## 8 Safety data

Conventional chemotherapeutic drugs such as paclitaxel, fluorouracil, tamoxifen, and raloxifene are a few commonly used chemo-preventive medications; but, besides their beneficial effect, such chemotherapy also poses several harmful effects (85). For this reason, over the past few years, the use of traditional medicinal plants for the treatment of various diseases has caught the attention of scientists. Medicinal plants are rich in bioactive components and phytochemicals and protect against many diseases without causing detrimental effects on the body’s organs. Such plants are highly advantageous for the innovation of numerous drugs with enormous benefits and lower undesirable effects. Isothiocyanates, especially sulforaphane, are highly effective against cancer and have been used as a medicine in China for thousands of years (86–88). Numerous studies proved that SFN can be used at various doses and in combination with other compounds without showing toxicity. A randomized controlled trial was designed to assess the safety and therapeutic effect of sulforaphane-rich drinks on thyroid hormonal status and thyroid autoimmune status. The results of the 12-week study concluded that the beverage containing on average of 40 µmol of SFN and 600 µmol of glucoraphanin per day was safe to consume (89).

## 9 Conclusion

Natural bioactive compounds currently have numerous applications in cancer chemotherapy. Furthermore, the vast spectrum of natural compounds provides important compounds for therapeutic refinement through molecular modifications. Many anticancer compounds are either natural products or substances derived from natural products. The conjugation of toxic natural products with monoclonal antibodies or with macromolecular carriers can lead to more precisely targeted therapies. As only a small percentage of higher plants have been systematically investigated, research on natural compounds as chemotherapeutic agents arouses further interest as well as multidisciplinary scientific collaborations. The results of preclinical studies showed that sulforaphane provides cancer protection through the alteration of various epigenetic and non-epigenetic pathways. The therapeutic limitations of the use of sulforaphane in cancer are represented by the complete lack of knowledge of the potential anticancer effects at the cellular level and the effects on the immune system, the lack of translational studies to establish the effective doses in humans, and the interactions with conventional chemotherapy medication. However, considering everything together, the results of preclinical and clinical studies point to new therapeutic perspectives towards the possible development of new sulforaphane-based anticancer drugs.

## Author contributions

All authors made a significant contribution to the work reported, whether that is in the conception, study design, execution, acquisition of data, analysis, and interpretation, or in all these areas, that is, revising or critically reviewing the article; giving final approval of the version to be published; agreeing on the journal to which the article has been submitted; and confirming to be accountable for all aspects of the work. All authors have read and agreed to the published version of the manuscript.
